# Engagement of Overexpressed Her2 with GEP100 Induces Autonomous Invasive Activities and Provides a Biomarker for Metastases of Lung Adenocarcinoma

**DOI:** 10.1371/journal.pone.0025301

**Published:** 2011-09-22

**Authors:** Toshi Menju, Shigeru Hashimoto, Ari Hashimoto, Yutaro Otsuka, Haruka Handa, Eiji Ogawa, Yoshinobu Toda, Hiromi Wada, Hiroshi Date, Hisataka Sabe

**Affiliations:** 1 Department of Thoracic Surgery, Graduate School of Medicine, Kyoto University, Kyoto, Japan; 2 Department of Molecular Biology, Hokkaido University Graduate School of Medicine, Sapporo, Japan; 3 Laboratory of Diagnostic Pathology, Kyoto University Hospital, Kyoto, Japan; Cornell University, United States of America

## Abstract

Overexpression of Her2/ErbB2/Neu in cancer is often correlated with recurrent distant metastasis, although the mechanism still remains largely elusive. We have previously shown that EGFR, when tyrosine-phosphorylated, binds to GEP100/BRAG2 to activate Arf6, which induces cancer invasion and metastasis. We now show that overexpressed Her2 in lung adenocarcinoma cells also employs GEP100. Like EGFR-GEP100 binding, this association is primarily mediated by the pleckstrin homology (PH) domain of GEP100 and Tyr1139/Tyr1196 of Her2. Tyr1139/Tyr1196 are autonomously phosphorylated, when Her2 is overexpressed. Accordingly, invasive activities mediated by the Her2-GEP100 pathway are not dependent on external factors. Blocking Her2-GEP100 binding, as well as its signaling pathway all inhibit cancer invasive activities. Moreover, our clinical study indicates that co-overexpression of Her2 with GEP100 in primary lung adenocarcinomas of patients is correlated with the presence of their node-metastasis with a statistical significance. Since the GEP100 PH domain interacts with both Her2 and EGFR, targeting this domain may provide novel cancer therapeutics.

## Introduction

The major cause of cancer-related death is the dissemination and distant metastasis of cancer cells. Overexpression of Her2/ErbB2/Neu, a member of the epidermal growth factor receptor (EGFR)-family tyrosine kinases, in malignant tumors is often correlated with recurrent distant metastasis [1], [2]. Cancer cells which overexpress Her2 generally exhibit highly migratory and invasive properties *in vitro* and *in vivo* [3], [4]. On the other hand, Her2 expression in cancer cells is inversely correlated with cancer-induced angiogenesis [5]. Therefore, overexpression of Her2 and its intracellular signaling appear to be directly related to the invasive and metastatic activities of cancer cells themselves. No direct ligand for Her2 *per se* has been identified, and overexpression of Her2 induces its homo-dimer/oligomer formation and its tyrosine phosphorylation, independent of ligand binding or oncogenic mutations [6]. Furthermore, Her2 expressed at moderate levels can be activated and tyrosine phosphorylated by heterodimerization with other EGFR-family receptors and stimulatation by their cognate ligands [7].

A small GTPase, Arf6, primarily regulates the recycling of plasma membrane components and plays pleiotropic roles [8], [9]. We have shown previously that Arf6 activity greatly contributes to the invasive and metastatic activities of breast cancer cells, when Arf6 is activated by GEP100/BRAG2 [10] and employs AMAP1/DDEF1/ASAP1 as a downstream factor [11], [12], [13]. EGF receptor (EGFR) is frequently overexpressed in many types of cancers, including breast cancer and lung cancer, and is highly implicated in their malignancy [14]. We have shown that EGFR, when activated by its ligand, recruits GEP100 and induces the invasion and metastasis of breast cancer cells [13]. Pathological analyses revealed that components of the EGFR-GEP100-Arf6-AMAP1 pathway are highly expressed in 40–80% of primary tumors of the human breast [12], [13]. We have recently indentified that this pathway acts to enhance the recycling of β1 integrin in order to enhance cancer cell invasive activities (Onodera *et al.*, submitted). Besides breast cancers, melanomas also utilize Arf6 activity for their invasion and metastasis [15].

GEP100 contains an incomplete IQ-motif, a pleckstrin homology (PH) domain and a Sec7 domain, which encodes guanine nucleotide exchanging factor (GEF) activity [10]. The PH domain was originally identified as a binding module to phophoinositides [16], while cognate ligands for most PH domains are still unidentified [17]. The PH domain of GEP100 does not show a notable specificity towards phophoinositides, and GEF activity of GEP100 was not stimulated by certain phophoinositides [8]. We have shown that this PH domain binds directly to phosphorylated Tyr1068 and Tyr1086 of EGFR [13]. It is hence important to clarify whether the GEP100 PH domain also binds to other tyrosine phosphorylated proteins to induce cancer invasion.

Human lung cancers are known to be more invasive, metastatic and lethal compared with other major epithelial tumors in clinical settings [18]. Her2 overexpression is detected in 15–40% of primary lung cancers [19], [20], [21], and correlates with the poor prognosis of the patients [22]. Randomized controlled trials using a humanized anti-Her2 monoclonal antibody, Trastuzumab, for patients with Her2-overexpressing lung cancers showed no significant survival benefits [23], contrary to the expectations from the preclinical studies [24]. Therefore, although possible mechanisms by which overexpressed Her2 induces cell migration and invasion have already been extensively studied [25], [4], further elucidation of Her2-mediated invasive/metastatic mechanisms in lung cancer are necessary for the future development of therapeutics for this disease. Non-small cell lung cancer is the most common subdivision of primary lung cancer, and is classified into several categories depending on the origin of the tumor, such as adenocarcinoma, squamous cell carcinoma and large cell carcinoma. Here, we show that Her2 overexpressed in lung adenocarcinoma cells employs GEP100 to activate the Arf6 pathway and induces their autonomous, stroma-independent invasive activities. Pathological analyses indicate that overexpression of both GEP100 and Her2 in lung adenocarcinoma specimens at the primary sites is statistically correlated with the presence of their node-metastasis. Such an autonomous invasive/metastatic property of Her2/GEP100-overexpressing lung cancers, as well as frequent existence of their node-metastasis already at the time of surgery, appears to be a part of the reason as to why Trastuzumab does not exhibit clear survival benefits in patients with this disease.

## Materials and Methods

### Ethics statement

This study was approved by the Kyoto University Hospital Institutional Review Board, and the written informed consent was obtained from all patients.

#### Complementary DNAs

cDNAs for GEP100, ARNO, and Her2 were described previously [13]. cDNA fragments each encoding the PH domain of GEP100 (631–742 amino acids) or ARNO (261–387 amino acids) were ligated into pGEX4T-2 to be in-frame to the COOH terminus of the GST-TK-tag, and expressed in *E.coli*, as described previously [13]. For mammalian expression, each cDNA was ligated into pEGFP (for the EGFP-tag), pcDNA3 HA (for the HA-tag), or pcDNA-myc (for the myc-tag). The YF mutants of Her2 were generated by the PCR-based mutagenesis method, in which tyrosines 1139, 1196, 1221, 1222 and 1248 were changed into phenylalanine, singly or in combination.

#### Accession numbers

Human GEP100, AB018306; human ARNO, X99753; human Her2, NM_004448; human AMAP1, NM_018482; and human Arf6, NM_001663.

#### Chemicals and antibodies

AG825 and EGF were purchased from Calbiochem and Sigma-Aldrich, respectively. Rabbit polyclonal antibody against GEP100 was as described previously [13]. Other antibodies were purchased from commercial sources: mouse monoclonal antibodies against hemaggultinin (HA)-tag (Babco), GFP-tag (Babco), Arf6 (Santa Cruz), AMAP1/ASAP1 (BD Biosciences), β-actin (Sigma), EGFR (TDL), Her2 (Neomarker), and Her3 (Neomarker); rabbit polyclonal antibodies against HA-tag (Babco), GFP-tag (Abcam), and Her2 (DAKO, for immunohistochemistry); rabbit monoclonal antibody against phospho-Her2 (Y1221/1222) (Cell Signaling). Donkey antibodies against rabbit and mouse IgGs, conjugated with horseradish peroxidase, were from Jackson ImmunoResearch Laboratories.

#### Cells

293T cells obtained from American Type Culture Collection were cultured in Dulbecco's modified Eagle's medium (DMEM) with 10% FCS (Hyclone). Human non-small cell lung cancer cell lines were obtained from and authenticated by American Type Culture Collection and RIKEN (Lu99, PC9 and PC14), and were cultured in RPMI-1640 medium with 10% FCS. All above cell lines were used in our experiments within 15 passages.

#### Transfection

cDNA transfections were performed by using Lipofectamine 2000 (Invitrogen) for H522 cells; and Polyfect (Qiagen) for 293T cells, according to the manufacturer's instructions. Transfected cells were incubated for 24 h with growth medium or in a starved condition before being subjected to analyses.

RNA interferrence was performed with 25 nM of duplex oligonucleotides using Lipofectamine 2000 (Invitrogen) and incubated for 48 h before being subjected to analyses. Oligonucleotides were synthesized by Japan BioServices (Saitama, Japan). Sequences used were as follows. GEP100: 5′-GTGAAATCACTGGCCGAGT-3′, Arf6: 5′-GCACCGCATTATCAATGACCG-3′, and AMAP1: 5′-GACCTGACAAAAGCCATTA-3′. The control irrelevant siRNA was 5′-GCGCGCTTTGTAGGATTCG-3′. Upon siRNA treatment, cell viability was measured using the 3-(4,5-dimethylthiazol-2-yl)-5-(3-carboxyphenyl)-2-(4-sulfophenyl)-2H-tetrazolium (MTS) assay, and ratios of the viability between the treated cells and the control are shown.

### Glutathione S-transferase (GST)–golgi-localized, γ-ear-containing, Arf-binding protein (GGA) pulldown, immunoprecipitation and immunoblotting

Arf6 activities were measured using GST-GGA, as previously described [26], by using 500 µg of cell lysates in each assay. Shortly, cells were transfected with Arf6-myc, HA-GEP100, and Her2-GFP, and the cells were incubated for 24 hours in serum-free DME medium. Cells were lysed in GGA3-buffer (50 mM Tris-HCl (pH 8.0), 100 mM NaCl, 10 mM MgCl_2_, 0.005% SDS, 0.05% sodium deoxycholate, 1% Triton X-100, 10% glycerol with 1 mM phenylmethylsulfonyl fluoride, 1 mM Na_3_VO_4_, and 5 µg/ml aprotinin). Each lysate was precleared by centrifugation after the incubation for 30 minutes with the glutathione magnetic beads (Thermo Scientific). Then, 500 ug of each clarified lysate was incubated with 20 ug of GST-GGA3 bound to glutathione magnetic beads at 4°C for 40 min. The beads were then washed three times with GGA3-buffer. Bound proteins were eluted into SDS-PAGE sample buffer. These samples were measured for the presence of Arf6 by Western blotting with the antibody against myc-tag. Total levels of Arf6-myc, HA-GEP100, and Her2-GFP were assayed by Western blotting of 20 ug of the precleared lysate.

For coprecipitation assays of GEP100 with Her2, cells were lysed in GGA3 buffer. 300 µg of cell lysates were then incubated with an anti-GEP100 polyclonal antibody, coupled to Protein G-magnetic beads (Millipore).

For *in vitro* protein binding assays, 25 µg of GST-fused PH domain, expressed in bacteria and purified on glutathione-beads, were incubated with 200 µg of cell lysates prepared in GGA3 buffer at 4°C for 1 h, and proteins co-precipitated with the beads were analysed by immunoblots, as described previously [13].

Immunoblotting analysis, coupled with SDS-PAGE, was performed as described previously [13].

#### Matrigel invasion

Matrigel chemoinvasion assay was performed with Biocoat Matrigel chambers (BD Biosciences), as described previously [11], in which 1×10^5^ cells were seeded on the upper wells. No chemoattractants or serum was added in the chambers. After incubation for 24 h, cells were fixed and stained with Diff-Quick solutions (Sysmex), and the number of cells that migrated-out to the lower surface of the membranes were scored. Data were collected from three independent experiments, each done in duplicate.

#### Pathology

All clinical specimens were from patients with primary lung adenocarcinomas, who underwent pulmonary resection at Kyoto University Hospital between May 2001 and September 2004. This study was approved by the Kyoto University Hospital Institutional Review Board, and the written informed consent was obtained from all patients. Pathological stage was evaluated according to the 6th version of international tumor, node, metastasis (TNM) staging system and the classification of the World Health Organization (WHO). Patients with pathological stage 3B or 4 were unexpectedly recognized during or after the surgical operation, or during the palliative surgery. Patient data were obtained from inpatient and outpatient medical records.

Immunohistochemical staining was performed on 4 µm-thick formalin-fixed paraffin-embedded sequential sections. Immunohistochemical staining against GEP100 or Her2 was performed by using the standard avidin-biotin-peroxidase complex (ABC) method, as described [12]. Each section was counterstained with hematoxylin. Two investigators independently scored the maximal intensity of tumors (HER2; 0∼3+, GEP100; 0∼2+).

#### Statistical analyses

Continuous variables in each experitment were analysed with ANOVA or Kruskal-Wallis test, and nominal variables were analysed with Fisher's exact test by using the statistics software, Graphpad Prism. The caluculated p-value was considered to be significant when less than 0.05.

## Results

### Binding of GEP100 with Her2

**Figure 1:**
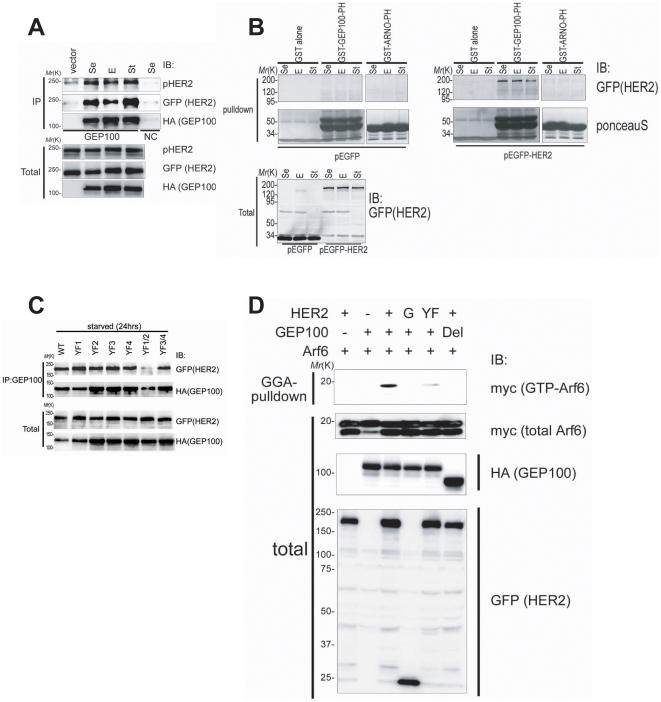
GEP100 associates with Her2 to induce Arf6 activation. (**A**) Co-precipitation of Her2-EGFP with HA-GEP100, expressed in 293T cells, and analysed by anti-GEP100 immunoprecipitation (IP) coupled with anti-GFP immunoblots (IB). Anti-GEP100 immunoprecipitants were also blotted by anti-phospho Her2 and an anti-HA antibody. Immunoprecipitation for the expression of Her2-EGFP without HA-GEP100 was included as a control (vector). Immunoprecipitation using non-immune serum was also included as a control (NC). (**B**) *In vitro* co-precipitation of Her2-EGFP (pEGFP-Her2) expressed in cells with the two indicated GST-tagged PH domains (GST-GEP100-PH/GST-ARNO-PH) or GST alone, analysed by glutathione-beads pulldown and anti-GFP immunoblots. GST-fusion proteins were visualized by Ponceau S. In **A** and **B**, cells were cultured with 10% FCS (Se), in the absence of serum for 24 h (St), or stimulated with 10 ng/ml EGF for 10 min after serum starvation for 24 h (E), prior to lysis. EGFP alone was included as a control (pEGFP). (**C**) Co-precipitation of HA-GEP100 with wild type Her2-EGFP (WT) or its mutants (YF1; 1139F, YF2; 1196F, YF3; 1221/1222F, YF4; 1248F, YF1/2; 1139/1196F, YF3/4; 1221/1222/1248F), analysed by anti-GEP100 immunoprecipitation and anti-GFP immunoblots. (**D**) Arf6-myc activities in cells expressing HA-GEP100 and Her2-EGFP or their mutants, measured by GST-GGA pulldown and anti-myc immunoblots. +, wild type; YF, Her2 1139/1196F mutant; Del, Sec7-deleted GEP100. EGFP alone was included as a control (G). In **A**–**D**, immunoblots of total cell lysates (10 µg) are also shown (Total).

Our previous analyses showed that GEP100 does not form a complex with Her2, which is expressed at a moderate level in MDA-MB-231 breast cancer cells [13]. Her2 is frequently overexpressed in many malignant cancers. We here examined whether overexpressed Her2 binds to GEP100. For this, we first expressed Her2, tagged with enhanced green fluorescence protein (EGFP) at the C-terminus, together with hemagultinnin (HA)-tagged GEP100 in 293T cells by cDNA transfection. Pull-down of HA-GEP100 using an anti-GEP100 antibody efficiently coprecipitated Her2-EGFP (Fig. 1A). Under these conditions, Her2-EGFP was heavily tyrosine phosphorylated, and this phosphorylation was not affected by the presence or absence of serum, nor by the addition of EGF (Fig. 1A). Binding of HA-GEP100 with Her2-EGFP was also unaffected by serum or EGF (Fig. 1A and B). We then performed an *in vitro* binding assay using affinity-purified glutathione-s-transferase (GST)-tagged GEP100 PH domain, and found that this domain alone binds to Her2-EGFP (Fig. 1B). As a control, we found that GST-tagged ARNO PH domain does not bind to Her2-EGFP (Fig. 1B).

We have shown previously that the GEP100 PH domain binds to phosphotyrosines [13]. Her2 overexpression induces autophosphorylation of its five major tyrosines, namely Tyr1139, Tyr1196, Tyr1221, Tyr1222 and Tyr1248 [14]. We then mutated these tyrosines into phenylalanines. We first found that none of the single mutations of Tyr1139 (1139F), Tyr1196 (1196F) and Tyr1248 (1248F) nor a double mutation of Tyr1221/1222 (1221/1222F) affect Her2 binding to GEP100 (Fig. 1C). However, we then found that simultaneous mutation of Tyr1139 and Tyr1196 (1139/1196F) abolished Her2 binding to GEP100 almost completely, while simultaneous mutation of Tyr1221, Tyr1222 and Tyr1248 (1221/1222/1248F) did not (Fig. 1C). These results suggest that Tyr1139 and Tyr1196 are each independently involved in GEP100 binding, similar to what we have observed in the case of EGFR binding to GEP100 [13]. The N-terminal sequences of Tyr1139 and Tyr1196 resemble each other, namely PQPEpY for Tyr1139 and ENPEpY for Tyr1196.

We then expressed Her2-EGFP or its mutants together with HA-GEP100 and Arf6-myc in 293T cells, and measured activation of Arf6-myc by use of the GST-GGA pulldown method [26]. We found that coexpression of Her2-EGFP with HA-GEP100 induces Arf6 activation, while coexpression of GFP with HA-GEP100 or coexpression of the YF1/2 mutant of Her2-EGFP with HA-GEP100 did not (Fig. 1D). No exogenous ligands were added in these experiments. Together with the results described above, these results collectively indicate that overexpressed Her2 physically associates with GEP100 to activate Arf6, which is primarily mediated by Tyr1139/1196 of Her2 and the PH domain of GEP100.

### Endogenous binding of Her2 with GEP100 in lung cancer cells

**Figure 2:**
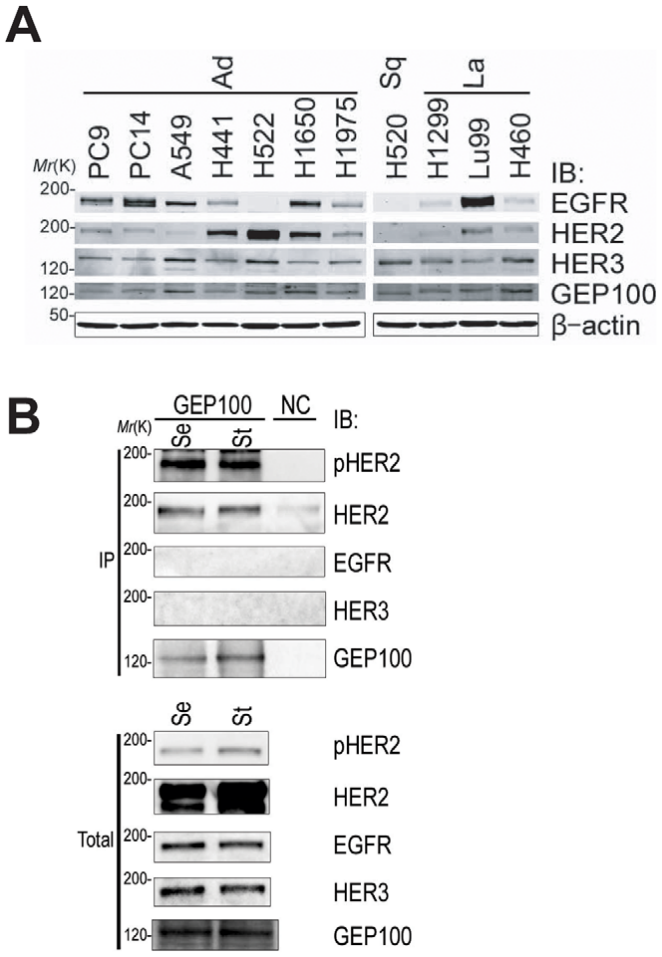
Endogenous binding of GEP100 with Her2 overexpressed in H522 lung adenocarcinoma cells. (**A**) Total lysates (30 µg) of 11 non-small cell lung cancer cell lines were immunoblotted with antibodies as indicated. (**B**) Co-precipitation of Her2 with GEP100 from H522 cell lysates (250 µg), analysed by anti-GEP100 immunoprecipitation, and the immunoblot for anti-GEP100, anti-Her2 (HER2), and anti-phospho Her2 (pHER2). Immunoblots using anti-EGFR (EGFR) and anti-Her3 (HER3) antibodies were also included. Cells were cultured in the presence of serum (Se), or starved for serum for 24 h (St), prior to lysis. Total, 20 µg of total lysates. Immunoprecipitation of cells using non-immune serum was used as a negative control (NC).

We next searched for non-small cell lung cancer cell lines that exclusively overexpress Her2. From the eleven human lung cancer cell lines we examined, including adenocarcinoma (Ad), squamous cell carcinoma (Sq) and large cell carcinoma (La), we found that H522 cells express Her2 at a very high level, while this cell line expresses only a small amount of Her3 and no detectable amount of EGFR (Fig. 2A). We found that Her2 is coprecipitated with an anti-GEP100 antibody and heavily tyrosine phosphorylated in H522 cell lysates, even when the cells are cultured in the absence of ligands or serum (Fig. 2B). H522 cells were derived from a lung adenocarcinoma. Mutations within the kinase domain of Her2 have been reported to occur in 1–10% of human lung adenocarcinomas [27], [28]. We however found no mutation within the Her2 kinase domain in H522 cells (data not shown).

### Inhibition of the Her2-GEP100 pathway blocks cancer invasion

**Figure 3:**
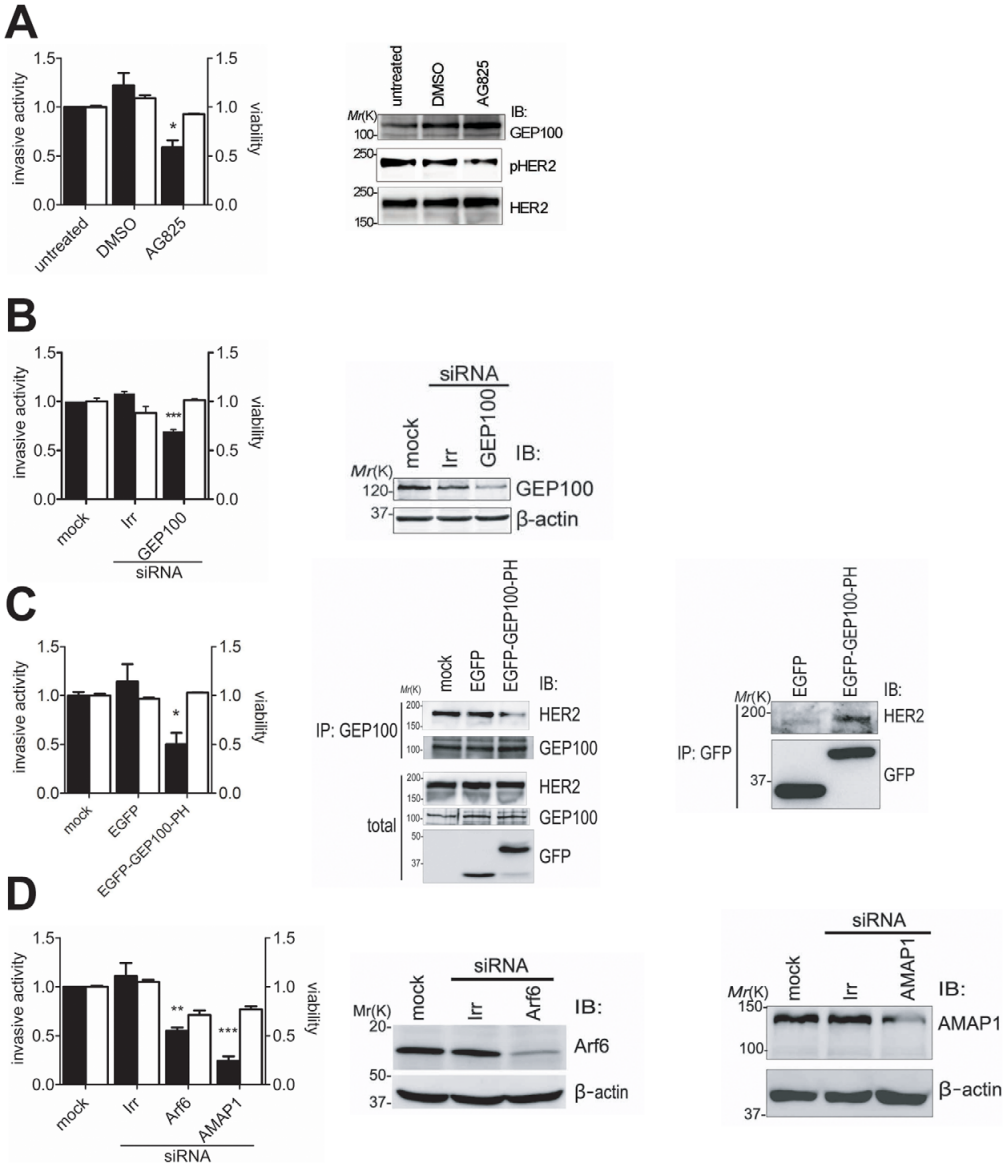
Inhibition of the Her2-GEP100 pathway blocks invasive activities of H522 cells. (**A**) Cells, untreated or treated with AG825 (10 µM) or DMSO (1%) for 72 h, were subjected to the Matrigel invasion assay, or their lysates (30 µg) were analysed by immunoblotting as indicated. Cell viability, measured by the MTS assay, is also shown. (**B**) Cells, untransfected (mock), or transfected with siRNA duplexes against GEP100 or irrelevant sequences (Irr), were subjected to the Matrigel invasion assay and the MTS assay; or were analysed for their expression of GEP100 by immunoblotting of the lysates (20 µg) using the antibody as indicated. β-actin immunoblots were included as controls. (**C**) Cells, expressing EGFP-tagged GEP100 PH domain (EGFP-GEP100-PH) or EGFP alone (EGFP), were subjected to the Matrigel invasion assay and the MTS assay. Inhibition of the intracellular association of GEP100 with Her2 by EGFP-GEP100-PH, as well as the association of EGFP-GEP100-PH with endogenous Her2, were confirmed by anti-GEP100 and anti-GFP immunoprecipitation coupled with anti-Her2 immunoblot, as indicated. IP, immunoprecipitant for total cell lysates (250 µg). Total, total cell lysates (30 µg). (**D**) Cells, untransfected (mock) or transfected with siRNA duplexes against Arf6, AMAP1, or with irrelevant sequences (Irr), were subjected to the Matrigel invasion assay and the MTS assay; or were analysed for their expression of Arf6 and AMAP1 by immunoblotting of the lysates using the antibodies as indicated. β-actin immunoblots were included as controls. In each bar chart, data of invasive activities (black columns) and cell viabilities (white columns) are presented as percentages calculated by normalizing the values obtained for the untreated cells (3A) which means native cells, or the mock cells (3B, 3C, and 3D) to which only transfection reagents were added as 1.0. Error bars show mean +/− s.e.m., n = 3. *, p<0.05; **, p<0.01; ***, p<0.001.

H522 cells have been shown to be invasive as assessed by the Matrigel invasion assay [29]. We first confirmed that treatment of H522 cells with AG825, an inhibitor of Her2 kinase activity, blocks their Matrigel invasion activity (Fig. 3A). Note that, however, AG825 even at 10 µM could only partially inhibit the kinase activity of Her2 in H522 cells, as assessed by the phosphorylation of Tyr1221/1222 (Fig. 3A), and inhibited Matrigel invasion activity only by about 60% (Fig. 3A). We next tested the effects of GEP100 knockdown. We tested several different sequences of GEP100 siRNAs, but all of them were not very effective in inhibiting protein expression of GEP100 in H522 cells (Fig. 3B). Under these conditions, GEP100 siRNAs inhibited their Matrigel invasion activity by about 40% (Fig. 3B). We then overexpressed the GFP-tagged GEP100 PH domain to block the endogenous binding of Her2 with GEP100. Although blockage by the GFP-GEP100 PH domain was not complete under this condition (Fig. 3C), the Matrigel invasion activity of H522 cells was inhibited by 50% (Fig. 3C). Matrigel invasion activities of H522 cells were unaffected by 0.1–10 ng/ml EGF (data not shown), and we did not add chemoattractants or serum throughout these Matrigel invasion assays.

We also sought to obtain evidence that Arf6 as well as its effector, AMAP1, are involved in the invasion activity of H522 cells. We found that Ar6 and AMAP1 siRNAs are effective in suppressing these protein expression in H522 cells (Fig. 3D), and the Matrigel invasion activity is inhibited by 50% and 70%, respectively (Fig. 3D). Note that unlike GEP100 siRNAs, AMAP1 siRNAs notably affected cell viability (Fig. 3D). Treatment of cells with Arf6 siRNA affected the viability of H522 cells more seriously, and we could not properly assess the invasion activity.

### High levels of GEP100 expression in Her2-overexpressing lung adenocarcinomas is correlated with node-metastasis

**Figure 4:**
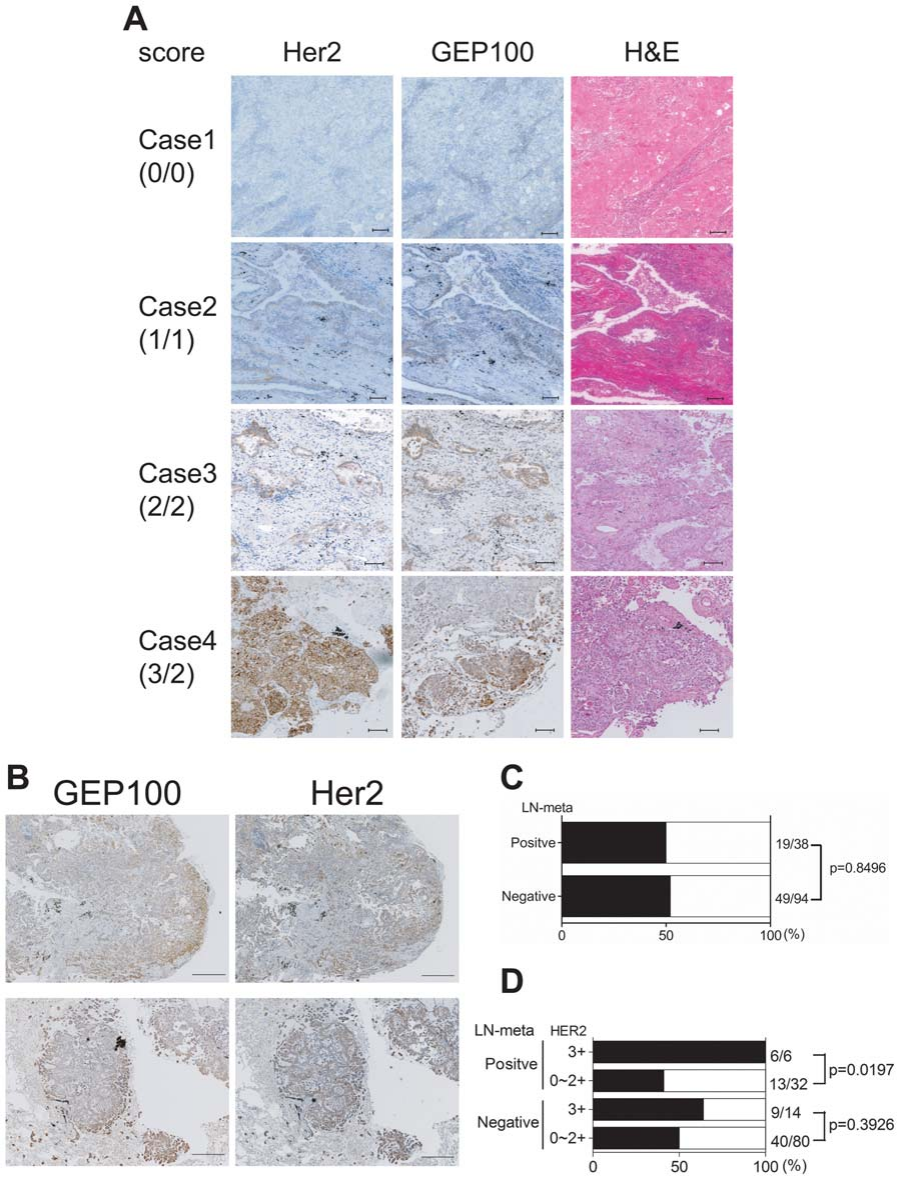
Immunohistochemistry of Her2 and GEP100 in adenocarcinoma lung tissue specimens. (**A**) Her2 and GEP100 were each stained in brown in sequential sections. Case 1, Her2 and GEP100 double negative; Case 2, Her2 score 1+ and GEP100 score 1+; Case 3, Her2 score 2+ and GEP100 score 2+; Case 4, Her2 score 3+ GEP100 and GEP100 score 2+. All sections were counterstained with hematoxylin and eosin (H&E) and representative figures are shown. Scale bars, 100 µm. (**B**) Representative figures of the peripheral expression of GEP100. Scale bars, 500 µm. (**C**) No correlation between strong expression of GEP100 (score 2+) *per se* with node-metastases. (**D**) A statistical correlation between double strong positive signal of GEP100 (score 2+) and Her2 (score 3+), and node-metastases. In **C** and **D**, solid bars mean GEP100 positive rate for strong expression (score 2+).

Adenocarcinoma is the major histological subtype of lung cancer, and rates of Her2 overexpression are significantly higher in adenocarcinomas than other histological types of lung cancer [30]. One hundred and thirty two specimens of lung adenocarcinoma were immunostained for Her2 and GEP100. Their maximum intensities was scored by two independent investigators (T.M. and Y.T.), in which scores were 0∼3+ for Her2 and 0∼2+ for GEP100. Table 1 shows characteristics of the patients, and Table 2 shows distribution of the scores. Representative staining images are shown in Fig. 4A. We used the same antibody against Her2 as used in the Herceptest (DAKO, A0485). Our results of Her2 staining were consistent with a previous report [31], in which the population of specimens scored 2+ and 3+ were approximately 40%. Among the 132 specimens, about 50% of them were strongly positive for GEP100 (score 2+). Notably, GEP100 expression within each cancer was heterogeneous, while Her2 expression was rather homogeneous (Fig. 4B). On the other hand, sites of maximum intensity of GEP100 staining were located mostly at peripheral areas of the cancer mass (Fig. 4B), consistent with a notion that invasion is primarily initiated by cells located at the periphery.

**Table 1:** Patient profiles.

Sex	M/F	63/69
Age	31–83 (mean 66)	
Smoking	current/ex/never	41/27/64
Differentiation	well/moderately/poorly	59/54/19
T-factor	T1/T2/T3/T4	82/34/7/9
N-factor	N0/N1/N2/N3	94/15/22/1
p-stage	1A/1B/2A/2B/3A/3B/4	67/22/5/6/21/7/4

**Table 2:** Summary of immunohistochemical analyses of surgical specimens of human lung adenocarcinoma.

		HER2					
		0	1+	2+	3+	Total	
GEP100	0	17	9	5	0	31	(23.5%)
	1+	8	15	5	5	33	(25%)
	2+	7	23	23	15	68	(51.5%)
	Total	32	47	33	20	132	
		(59.8%)		(40.2%)			

The expression pattern of Her2 and GEP100 in 132 samples of lung adenocarcinoma were determined and summarized.

Throughout these specimens, strong expression of GEP100 (score 2+) *per se* was not correlated with node-positive status (metastatic, 19/38 (50.0%) vs. non-metastatic, 49/94 (52.1%); p = 0.8496) (Fig. 4C). However, in the node-positive cases with Her2-overexpression (score 3+), all of them also overexpressed GEP100 (score 2+). On the other hand, in the node-positive cases with lower Her2 expression (score 0∼2+), less than 50% overexpressed GEP100 (Her2 score 3+ & GEP100 score 2+, 6/6 (100.0%) vs. Her2 score 0∼2+ & GEP100 score 2+, 13/32 (40.6%); p = 0.0197) (Fig. 4D). In the node-negative cases, GEP100 expression was not correlated with their Her2 status (Fig. 4D). Therefore, strong co-expression of GEP100 and Her2 in lung adenocarcinomas at the primary sites is significantly correlated with presence of their node-metastases.

## Discussion

Her2 overexpression is a major risk factor for various types of cancer [5], [32], [33]. Intracellular signaling pathways of Her2 have been extensively studied, and Her2 may promote invasive and metastatic activities through Ras- and Rho-family small GTPases [34], [35], PI3K/Akt pathway [36], MMP [25], and also miR21 [37]. Here we show that overexpressed Her2 activates another small GTPase, Arf6, via its association with GEP100, and induces cancer cell invasion. Our results indicate that simultaneous overexpression of GEP100 and Her2 in primary lung adenocarcinomas is statistically correlated with the presence of node-metastases. We hence propose that overexpression of both GEP100 and Her2 provides a biomarker predictive for the distant metastases of lung adenocarcinomas.

GEP100 expression was heterogeneous even within a single lesion of lung adenocarcinoma, while Her2 showed more homogeneous expression. On the other hand, GEP100 expression was predominantly observed at peripheries of the cancer mass. Therefore, expression of GEP100 in cancer cells does not seem to be a primary result of genome alterations that caused the cells to become cancerous, but may be inducible even in malignant cancer cells. Therefore, identification of factors and conditions that affect GEP100 protein expression, within cancer cells and the microenvironment, will be important for understanding the mechanism that determines whether lung adenocarcinoma cells become invasive and metastatic.

Resistance against or inefficacy of Trastuzumab, used as a single agent, occurs frequently in breast cancer and lung cancer [38], [39]. One explanation for this would be the incomplete inhibition of overexpressed Her2 by Trastuzumab, which may then cause lateral activation of residual Her2 molecules through their heterodimerization with other EGFR-family members [40]. The GEP100 PH domain binds both to EGFR and Her2. Moreover, engagement of GEP100 with these growth factor receptor tyrosine kinases does not occur constitutively, but appears to occur only during specific cellular conditions such as movement [13]. Therefore, targeting this PH domain appears to be highly advantageous, not only since it inhibits both Her2 and EGFR signaling for invasion, but since it may exhibit little side effects on cellular mechanisms other than migration. Small inhibitors targeting this domain might also be effective when used in combination with Trastuzumab, perhaps by compensating for the inefficacy of Trastuzumab-based therapy.
